# Methadone but not Morphine Inhibits Lubiprostone-Stimulated Cl^−^ Currents in T84 Intestinal Cells and Recombinant Human ClC-2, but not CFTR Cl^−^ Currents

**DOI:** 10.1007/s12013-012-9406-6

**Published:** 2012-08-24

**Authors:** John Cuppoletti, Jayati Chakrabarti, Kirti Tewari, Danuta H. Malinowska

**Affiliations:** Department of Molecular and Cellular Physiology, University of Cincinnati College of Medicine, 231 Albert Sabin Way, Cincinnati, OH 45267-0576 USA

**Keywords:** ClC-2 Cl^−^ channel, Methadone, Lubiprostone, Morphine, T84 cells, Prostone

## Abstract

In clinical trials, methadone, but not morphine, appeared to prevent beneficial effects of lubiprostone, a ClC-2 Cl^−^ channel activator, on opioid-induced constipation. Effects of methadone and morphine on lubiprostone-stimulated Cl^−^ currents were measured by short circuit current (Isc) across T84 cells. Whole cell patch clamp of human ClC-2 (hClC-2) stably expressed in HEK293 cells and in a high expression cell line (HEK293EBNA) as well as human CFTR (hCFTR) stably expressed in HEK293 cells was used to study methadone and morphine effects on recombinant hClC-2 and hCFTR Cl^−^ currents. Methadone but not morphine inhibited lubiprostone-stimulated Isc in T84 cells with half-maximal inhibition at 100 nM. Naloxone did not affect lubiprostone stimulation or methadone inhibition of Isc. Lubiprostone-stimulated Cl^−^ currents in hClC-2/HEK293 cells, but not forskolin/IBMX-stimulated Cl^−^ currents in hCFTR/HEK293 cells, were inhibited by methadone, but not morphine. HEK293EBNA cells expressing hClC-2 showed time-dependent, voltage-activated, CdCl_2_-inhibited Cl^−^ currents in the absence (control) and the presence of lubiprostone. Methadone, but not morphine, inhibited control and lubiprostone-stimulated hClC-2 Cl^−^ currents with half-maximal inhibition at 100 and 200–230 nM, respectively. Forskolin/IBMX-stimulated hClC-2 Cl^−^ currents were also inhibited by methadone. Myristoylated protein kinase inhibitor (a specific PKA inhibitor) inhibited forskolin/IBMX- but not lubiprostone-stimulated hClC-2 Cl^−^ currents. Methadone caused greater inhibition of lubiprostone-stimulated currents added before patching (66.1 %) compared with after patching (28.7 %). Methadone caused inhibition of lubiprostone-stimulated Cl^−^ currents in T84 cells and control; lubiprostone- and forskolin/IBMX-stimulated recombinant hClC-2 Cl^−^ currents may be the basis for reduced efficacy of lubiprostone in methadone-treated patients.

## Introduction

Lubiprostone in IRB-approved clinical trials has been shown to ameliorate opioid-induced constipation [1, 2]. However, post hoc subgroup analysis of clinical trial results [3] suggested methadone, but not morphine, attenuated/prevented the beneficial effects of lubiprostone. Lubiprostone stimulates T84 cell short circuit current (Isc) [4–6]. The present study tested the following hypothesis: methadone, but not morphine, attenuates lubiprostone-stimulated Cl^−^ transport in T84 cells. Lubiprostone-stimulated Isc in T84 cells is thought to be due to human ClC-2 (hClC-2) from T84 cell hClC-2 knockdown experiments [7]. However, lubiprostone activated not only A6 cell ClC-2 at low concentrations (<100 nM), but also A6 cell CFTR at higher concentrations (50 times higher concentrations than required to activate ClC-2) [8]. CFTR has also been suggested to be solely responsible for lubiprostone stimulation of T84 Isc [5, 6]. Therefore, the effects of methadone and morphine on hClC-2; and human CFTR (hCFTR) Cl^−^ currents were also determined. To measure hClC-2 and hCFTR Cl^−^ currents, whole cell patch clamp studies of the effects of lubiprostone, methadone, and morphine were carried out on previously used [4, 8–10] HEK293 cells stably transfected with either recombinant hClC-2 or recombinant hCFTR. A modified HEK293 cell line (HEK293EBNA) constitutively expressing Epstein Barr Virus nuclear antigen 1 (EBNA-1) to allow for high copy episomal replication of oriP containing plasmids, such as pCEP4, was stably transfected with hClC-2 in pCEP4 and also used. This cell line expressed higher control time-dependent, voltage-activated hClC-2 Cl^−^ currents, unlike the previously used [4, 9, 10] hClC-2-transfected HEK293 cells did, wherein lower control Cl^−^ currents and infrequent time-dependence and voltage-activation were evident.

Opioid receptors are present on the nerves of the gastrointestinal tract [11, 12], but not all opioid effects on ion channels involve these receptors. Thus, l-type, but not T-type Ca^2+^ channels [13], several K^+^ channels [14, 15] and the hERG K^+^ channel [16] have been shown to be differentially inhibited by methadone over morphine, in a manner independent of mu receptor occupation. Inhibition of hERG by methadone is problematic, as methadone treatment may lead to cardiotoxicity (long QT syndrome) [16, 17]. Methadone inhibition of hERG has been suggested to be caused possibly by direct binding to the hERG channel, perhaps at the voltage sensor [16].

Although there have been no previous reports of inhibition of Cl^−^ transport in T84 cells as measured by Isc by any opioids, it appeared reasonable, in the face of the apparent failure of lubiprostone to ameliorate methadone-induced constipation [3], and evidence for non-opioid receptor mechanisms of methadone inhibition of some ion channels [13–16], to test whether methadone and morphine had any effect on Isc in T84 cells.

ClC-2 is a time-dependent, voltage-activated Cl^−^ channel exhibiting inward rectification [8, 18–21] and is inhibited by CdCl_2_ [18–21]. hClC-2 activation also occurs with forskolin/IBMX in a myristoylated PKI-sensitive manner [10] at two sites identified by site-directed mutagenesis [10]. The present studies of opioid effects on control hClC-2 Cl^−^ currents and forskolin/IBMX activation of hClC-2 were also undertaken to determine whether opioid effects were limited to lubiprostone activation, or were rather a general effect on ClC-2. The present study of methadone and morphine effects on Cl^−^ currents in T84 intestinal cells and on recombinant hClC-2 Cl^−^ currents might explain the lack of effectiveness of lubiprostone in a clinical trial on opioid-induced constipation in patients on methadone, but not morphine therapy [3].

## Materials and Methods

Human ClC-2- and hCFTR-transfected HEK293 cells, T84 cells, culture conditions, patch clamp, and Isc methods were as described in [4].

### Materials

Lubiprostone and DMSO were obtained from R-Tech Ueno, Japan. Methadone hydrochloride, morphine sulfate, and naloxone hydrochloride were obtained from Sigma-Aldrich (St. Louis, MO). Forskolin, 7-deacetyl-7-[*O*-(*N*-methylpiperazino)-γ-butyryl]-dihydrochloride and myristoylated PKA inhibitor 14–22 amide cell permeant (mPKI) were from EMD Millipore-Calbiochem (Billerica, MA). 1-Ethyl-2-benzimidazolinone (1-EBIO) and isobutylmethylxanthine (IBMX) were purchased from Tocris Cookson (Ellisville, MO). Borosilicate glass (no. 7052) was obtained from Garner Glass (Claremont, CA). MEM, heat-inactivated horse serum, all supplements, G418, hygromycin, DMEM/Ham’s F12, heat-inactivated FBS, and Lipofectamine were from InVitrogen (Eugene, OR). HEK293EBNA cells, DMEM, and FCS were obtained from ATCC (Manassas, VA). Snapwell permeable supports were from Corning (Corning, NY). Lubiprostone, forskolin/IBMX, and 1-EBIO were dissolved in DMSO. DMSO was always kept at or below 0.2 %. Methadone, morphine, and mPKI were dissolved in water.

### Cell Culture

T84 cells were grown in DMEM/Ham’s F-12 medium with 6 % heat-inactivated FBS, 15 mM HEPES, 14.3 mM NaHCO_3_, 100 U/ml penicillin, and 100 μg/ml streptomycin sulfate and then grown to confluence on 1.13 cm^2^ Snapwell permeable supports.

Human ClC-2-transfected and hCFTR-transfected HEK293 cells were grown in MEM supplemented with 5 % heat-inactivated horse serum, 0.1 mM nonessential amino acids, 2 mM l-glutamine, 1 mM sodium pyruvate, 100 U/ml penicillin, 100 μg/ml streptomycin sulfate, and 300 μg/ml G418 and 100 μg/ml hygromycin, respectively. These two stably transfected cell lines have been extensively characterized and used in our previous studies [4, 9, 10]. In order to examine time-dependent, voltage-activated hClC-2 Cl^−^ currents in the absence of activators, a stable cell line greatly overexpressing hClC-2 was made using HEK293EBNA cells [22–27]. These cells have been transformed to constitutively express the EBNA-1. This allows for high copy episomal replication of oriP containing plasmids such as pCEP4. Recombinant hClC-2 was subcloned into the pCEP4 vector and transfected into HEK293EBNA cells using Lipofectamine (Invitrogen). Stable transformants were selected with 100 μg/ml hygromycin. HEK293EBNA cells were grown in DMEM containing 10 % FCS and 0.25 mg/ml G418.

### Short-circuit Current Measurements

The EasyMount Ussing chamber system (8 chambers) with VCCMC8 8-channel current–voltage (I–V) clamps from Physiologic Instruments (San Diego, CA) was used for Isc measurements across confluent T84 cell monolayers as previously described [4]. Transepithelial resistance of T84 cells was monitored with an EVOM epithelial volt ohm meter (World Precision Instruments). Cells were used when the transepithelial resistance of the monolayer was >1,200 Ω. Solutions were continuously gassed with 95 % O_2_–5 % CO_2_, also providing stirring, and the temperature was held constant at 37 °C with a heating block. The clamps were connected to Acquire & Analyze software (Physiologic Instruments) for automatic data collection from all eight of the Ussing chambers. Ag/AgCl reference electrodes were used for measuring transepithelial voltage and passing current. The basolateral membrane bath solution contained (in mM) 120 NaCl, 25 NaHCO_3_, 3.3 KH_2_PO_4_, 0.8 K_2_HPO_4_, 1.2 MgCl_2_, 1.2 CaCl_2_ (pH 7.4), and 10 mM glucose. The apical membrane bath solution was identical, except that the Cl^–^ concentration was reduced by substituting sodium gluconate for NaCl and CaCl_2_ was increased to 4 mM as previously described [4, 28] because of Ca^2+^ chelation by gluconate. Free [Ca^2+^] of the gluconate medium was calculated to be 1.2 mM using the Cabuf program (ftp://ftp.cc.kuleuven.ac.be/pub/droogmans/cabuf.zip) as was used previously [29]. 10 mM mannitol was used instead of glucose to ensure the absence of any Na^+^-glucose cotransport. To remove constraints on apical membrane Cl^−^ currents, 300 μM 1-EBIO, a Ca^2+^-activated K channel activator [30] was added to the basolateral bath solution and allowed to equilibrate.

### Patch Clamp Measurement of Whole Cell Cl^–^ Currents

Patch clamp and analytic methods were described previously [4]. Two voltage-clamp pulse protocols were used. For hClC-2-transfected and hCFTR-transfected HEK293 cells, currents were elicited by voltage-clamp pulses between −140 and +40 in 20-mV increments from a holding potential of −30 mV, and 200 ms recordings were made. For hClC-2-transfected and mock-transfected HEK293EBNA cells, currents were elicited by voltage-clamp pulses between −160 and +40 mV in 20-mV increments from a holding potential of −30 mV, and 1,500 ms recordings were made. For both protocols, current values were taken at 200 ms. The bath (external) solution contained (in mM) 140 tetraethylammonium Cl, 1 MgCl_2_, 2 CaCl_2_, and 10 HEPES (pH 7.4). The pipette (internal) solution contained (in mM) 115 tetraethylammonium Cl, 2 MgCl_2_, 5 EGTA, and 10 HEPES (pH 7.4). Pipettes were prepared from borosilicate glass and pulled by a two-stage Narishige puller to produce 1–1.5-MΩ resistance. Data were acquired with an Axopatch CV-4 headstage, a Digidata 1200 digitizer, and an Axopatch 1D amplifier. Data were analyzed using pClamp 6.04 (Axon Instruments, Union City, CA), Microsoft Excel, and Origin software (OriginLab, Northampton, MA). Cl^−^ currents were all measured at 200 ms and normalized to capacitance.

### Statistics

Statistical significance between two means was calculated using the Student’s *t* test. Significance was at *P* < 0.05 or less. In Fig. 3b, using Origin 5.0 Professional, the data were fit using a modified Michaelis–Menten hyperbolic function as previously described [4]. The equation used was Δ*I* = Δ*I*_max_ × [lubi]/(EC_50_ + [lubi]), where Δ*I*_max_ is the maximum change in *I*, and EC_50_ is [lubi] required for half-maximal response. As change in *I* was measured, the analysis was constrained to 0. All other graphs were plotted as mean ± SEMs joined by lines. Half-maximal inhibitory concentrations for methadone were estimated from the values at the control (no methadone) minus the values at the maximal concentration of methadone divided by two.

## Results

### Effect of Selected Concentrations of Methadone and Morphine on Lubiprostone-stimulated Cl^−^ Currents in T84 Cells and Effect of Naloxone

**Fig. 1:**
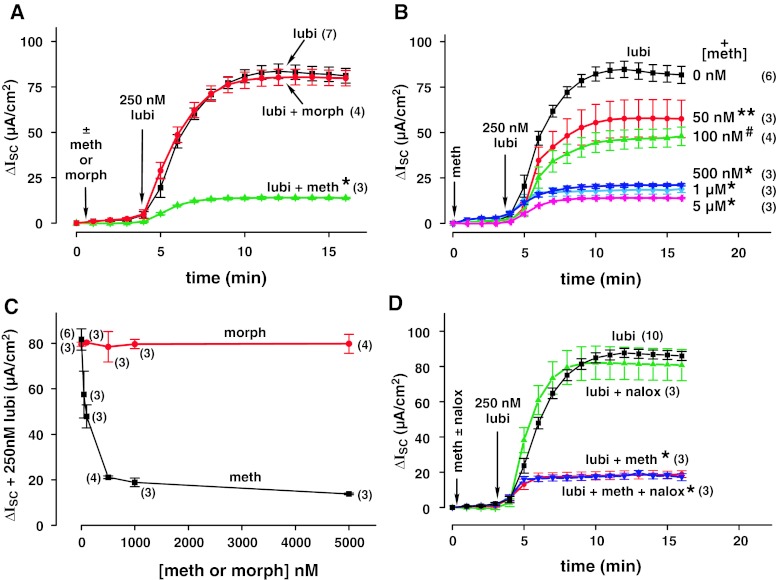
Effects of methadone and morphine on lubiprostone-stimulated Isc across T84 cell cultures and effect of naloxone. **a** T84 cells were mounted in an Ussing chamber under short circuit conditions, and first treated with 300 μM 1-ethyl-2-benzimidazolinone (1-EBIO) and then with either 5 μM morphine, 5 μM methadone, or no addition. 250 nM lubiprostone was then added, and Isc was measured. Mean ± SEM are *plotted*. Number of filters (*n*) measured under each condition is indicated. *P < 0.0005 versus lubiprostone alone. **b** Indicated concentrations of methadone were added before treatment with lubiprostone. Mean ± SEM are *plotted*; *n* is indicated. *P < 0.0005 methadone versus lubiprostone; #*P* < 0.001 methadone versus lubiprostone; **P < 0.05 methadone versus lubiprostone. **c** Effects of selected concentrations of methadone and morphine on lubiprostone-stimulated Isc are shown. Isc values *plotted* were taken at 16 min after start of the experiment, and 12 min after addition of lubiprostone. Mean ± SEM are *plotted*; *n* is indicated. **d** T84 cells were mounted in an Ussing chamber under short circuit conditions, and first treated with 300 μM 1-EBIO and then with nothing, followed by 10 μM naloxone, 1 μM methadone, or 10 μM naloxone plus 1 μM methadone. 250 nM lubiprostone was then added to all, and Isc was measured. Data are *plotted* as mean ± SEM and *n* is indicated. *P < 0.0005 versus lubi ± naloxone

The effects of methadone and morphine on lubiprostone-stimulated Isc in T84 cells were determined. The results are shown in Fig. 1. Prior addition of 5 μM morphine had no effect on 250 nM lubiprostone-stimulated Isc, but prior addition of 5 μM methadone caused major (83.1 %) inhibition of lubiprostone-stimulated Isc (Fig. 1a). The effect of selected concentrations of methadone on lubiprostone-stimulated Isc is shown in Fig. 1b, and the methadone and morphine concentration response curves are shown in Fig. 1c. Morphine had no effect at any concentration tested. However, methadone inhibited the lubiprostone-stimulated Isc in a concentration-dependent manner with half-maximal inhibition of Isc at 100 nM.

No evidence for mu receptors on intestinal cells was found by several investigators [11, 12, 31, 32], but in one study, evidence was found for mu receptors on human colonocytes [33]. Therefore, the effect of the nonspecific opioid receptor antagonist, naloxone, on methadone inhibition of Cl^−^ currents (Isc) in T84 cells was examined. As shown in Fig. 1d, 250 nM lubiprostone-stimulated Isc across T84 cells. Prior addition of 10 μM naloxone alone had no effect, while prior addition of 1 μM methadone was inhibitory. Addition of 10 μM naloxone had no effect on methadone inhibition of lubiprostone-stimulated Isc.

### Effects of Methadone and Morphine on Lubiprostone-stimulated and Forskolin/IBMX-stimulated Cl^−^ Currents in hClC-2-expressing HEK293 Cells

**Fig. 2:**
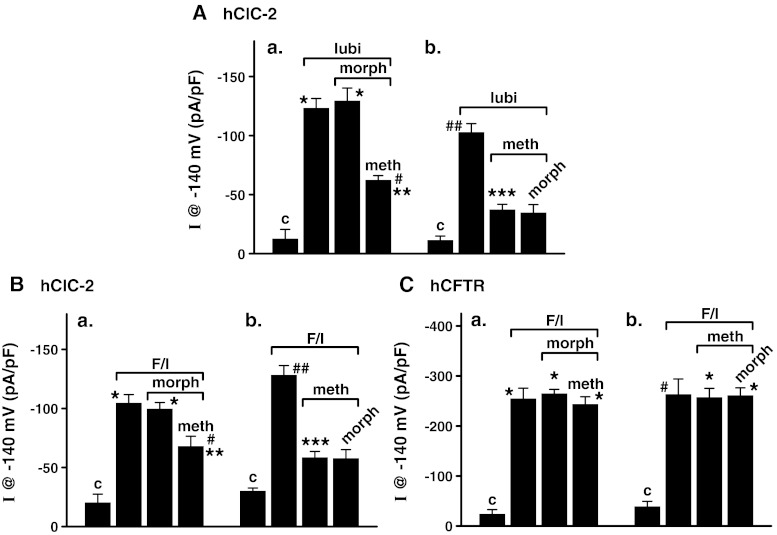
Effects of methadone and morphine on **A** lubiprostone-stimulated hClC-2 Cl^−^ currents; **B** forskolin/IBMX-stimulated hClC-2 Cl^−^ currents, and **C** forskolin/IBMX-stimulated CFTR Cl^−^ currents. HEK293 cells expressing hClC-2 or hCFTR were used. Cl^−^ currents were measured at −140 mV, 200 ms, and normalized to capacitance. In **A**, hClC-2-transfected HEK293 cells were treated with (*a*) 100 nM lubiprostone, followed by 100 nM morphine and then by 1 μM methadone or with (*b*) 100 nM lubiprostone, followed by 1 μM methadone and then by 100 nM morphine. Data are *plotted* as mean ± SEM (*n* = 3). In (*a*) *P < 0.001 versus control; #*P* < 0.02 versus lubi + morph; **P < 0.01 versus lubi; in (*b*) ##*P* < 0.005 versus control; ***P < 0.005 versus lubi. In **B**, hClC-2-transfected HEK293 cells were treated with (*a*) 5 μM forskolin/20 μM IBMX, followed by 100 nM morphine and then 1 μM methadone or with (*b*) 5 μM forskolin/20 μM IBMX followed by 1 μM methadone and then 100 nM morphine. Data are presented as mean ± SEM (*n* = 3). In (*a*) *P < 0.001 versus control; #*P* < 0.025 versus F/I & F/I + morph; **P < 0.01 versus control; in (b) ##*P* < 0.0005 versus control & F/I + meth; ***P < 0.01 versus control. In **C**, recombinant hCFTR-transfected HEK293 cells were treated with (*a*) 5 μM forskolin/20 μM IBMX followed by 100 nM morphine and then 1 μM methadone or with (*b*) 5 μM forskolin/20 μM IBMX followed by 1 μM methadone and then 100 nM morphine. Data are *plotted* as mean ± SEM (*n* = 4). In (*a*) *P < 0.0005 versus control; in (*b*) *P < 0.0005 versus control, and #*P* < 0.0025 versus control. Control (*c*) is the Cl^−^ current without lubiprostone being added

The effects of methadone and morphine, together and separately, on lubiprostone-stimulated hClC-2 Cl^−^ currents were next determined. Cl^−^ currents in hClC-2-transfected HEK293 cells were measured in cells without lubiprostone (control), cells treated with 100 nM lubiprostone, and with either 1 μM methadone followed by 100 nM morphine or the reverse 100 nM morphine followed by 1 μM methadone. As shown in Fig. 2a, 100 nM lubiprostone-stimulated hClC-2 Cl^−^ currents and 100 nM morphine had no effect on the currents. Subsequent addition of 1 μM methadone inhibited lubiprostone-stimulated Cl^−^ currents. Also as shown in Fig. 2a, addition of 1 μM methadone inhibited lubiprostone-stimulated Cl^−^ currents and subsequent addition of 100 nM morphine had no effect. The effects of 100 nM morphine and 1 μM methadone were also studied on hClC-2 Cl^−^ currents activated by 5 μM forskolin/20 μM IBMX, an alternative means of activation of hClC-2 blocked by mPKI [4, 10]. As shown in Fig. 2b, forskolin/IBMX activated hClC-2 Cl^−^ currents, and these currents were not affected by morphine, but inhibited by methadone, whether added after or before morphine. Thus, methadone appears to inhibit hClC-2, regardless of the method of activation, whether by lubiprostone or forskolin/IBMX. Methadone, but not morphine, whether added together or separately, inhibited both lubiprostone- and forskolin/IBMX-stimulated Cl^−^ currents in hClC-2-transfected HEK293 cells. Methadone thus apparently inhibits the hClC-2 Cl^−^ channel by a mechanism independent of lubiprostone, per se.

### Effects of Methadone and Morphine on Forskolin/IBMX-stimulated hCFTR Cl^−^ Currents

CFTR is also found in the intestine, and in one study was not activated by lubiprostone [4]. In other studies, CFTR appeared to be activated as well [5, 6, 8]. Therefore, the effects of 100 nM morphine and 1 μM methadone were also studied on Cl^−^ currents in HEK293 cells expressing recombinant hCFTR [4] after activation by 5 μM forskolin/20 μM IBMX. As shown in Fig. 2c, hCFTR Cl^−^ channel activity activated by forskolin/IBMX was not inhibited by methadone or morphine.

### Cl^−^ Currents Expressed in hClC-2-transfected HEK293EBNA Cells are Time-dependent, Voltage-activated, and Inhibited by CdCl_2_

**Fig. 3:**
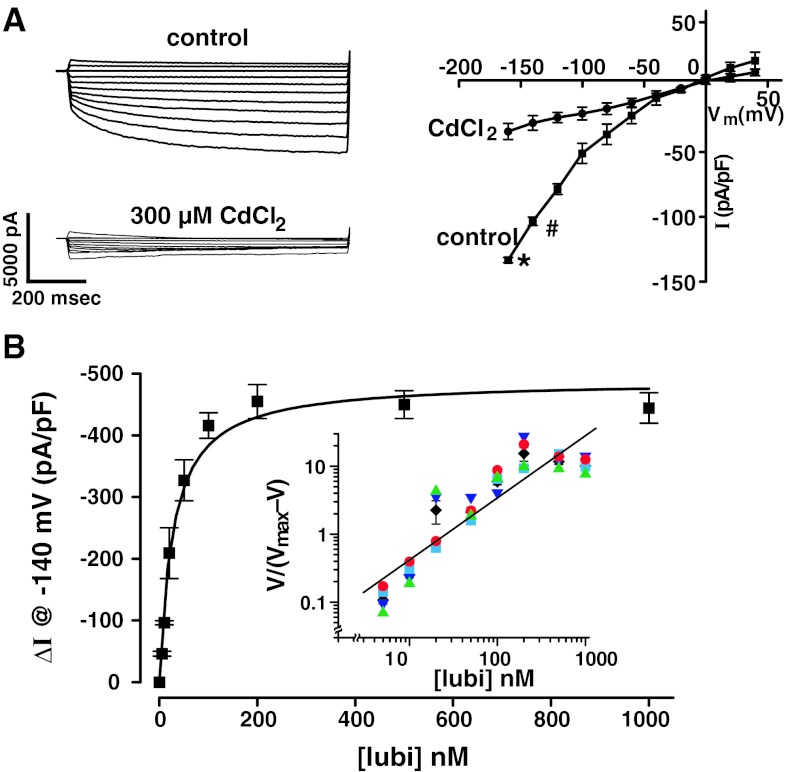
Cl^−^ currents expressed in hClC-2-transfected HEK293EBNA cells are time dependent and voltage activated: effects of **a** CdCl_2_ and **b** lubiprostone. Cl^−^ currents were measured in recombinant hClC-2-transfected HEK293EBNA cells by whole cell patch clamp. **a** Representative current recordings are shown for hClC-2-transfected cells before and after addition of 300 μM CdCl_2_ (cell capacitance = 31.6 pF) and corresponding I/V curves (*I* at 200 ms), *plotted* as mean ± SEM (*n* = 3) are also shown. *P < 0.005 and #*P* < 0.001 versus respective +CdCl_2_. At −120 and −100 mV, *P* < 0.0005 and *P* < 0.025, respectively, versus +CdCl_2_. In **b**, hClC-2 Cl^−^ current recordings were made at defined lubiprostone concentrations. *I* at −140 mV, 200 ms, normalized to capacitance (pA/pF) was *plotted* versus lubiprostone concentration. Data are *plotted* as mean ± SEM, *n* = 4 cells. *Curve* was fitted (using Origin) with a modified Michaelis–Menton equation: EC_50_ = 28.2 ± 2.2 nM (4) and the *V*_max_ = 489.8 ± 22.9 pA/pF (4). *Inset* shows the *Hill plot* of the data for the four cells, each a different *color*, (in *black* are means ± SEM for comparison, but not used for *Hill plot* calculations). Hill coefficient = 0.91 ± 0.02 (4), *R* = 0.96 ± 0.09 (8), and *P* = 0.000183 or *P* < 0.0005

**Fig. 4:**
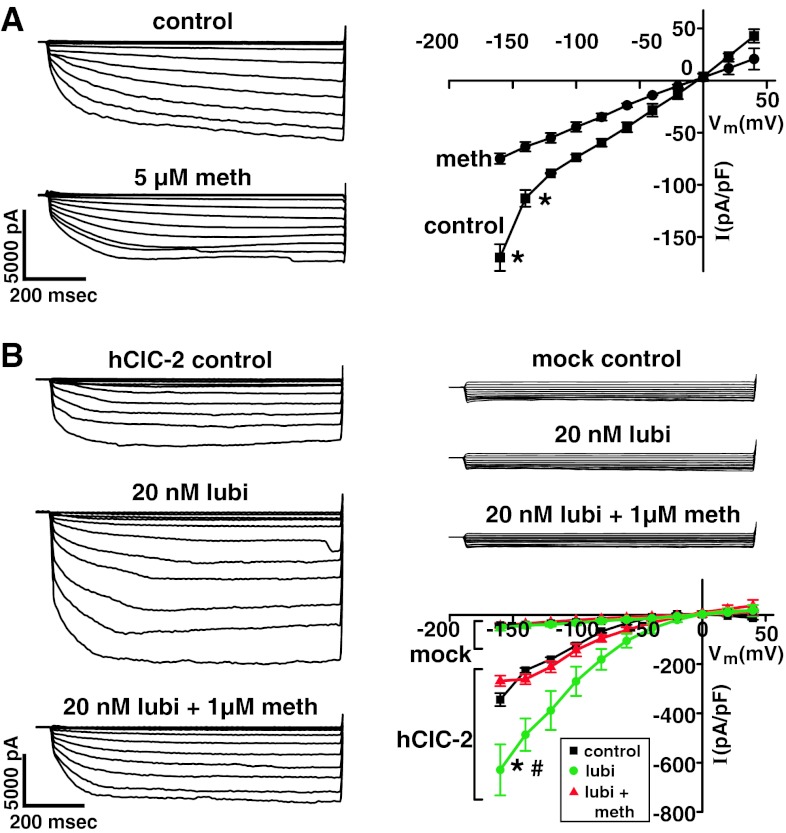
Effect of methadone on **a** control and **b** lubiprostone-stimulated Cl^−^ currents in hClC-2- and mock-transfected HEK293EBNA cells. Cl^−^ currents were measured in recombinant hClC-2- and mock-transfected HEK293EBNA cells by whole cell patch clamp. **a** Typical Cl^−^ current recordings before (control) and after addition of 5 μM methadone are shown in hClC-2-transfected HEK293EBNA cells (cell capacitance = 30.2 pF). Also shown is the I/V *curve* (*I* at 200 ms) with 1 μM methadone, plotted as mean ± SEM (*n* = 3). *P < 0.01 versus meth and at −120, −100, and −60 mV *P* < 0.01 versus meth; at −80 mV *P* < 0.005. **b** Typical Cl^−^ current recordings before (control) and after 20 nM lubiprostone, followed by 1 μM methadone are shown in hClC-2-transfected and mock-transfected HEK293EBNA cells, with cell capacitances = 21.2 and 28.8 pF, respectively. Corresponding I–V *curves* (*I* at 200 ms) are also shown *plotted* as mean ± SEM (*n* = 3). *P < 0.025 versus mock-transfected; #*P* < 0.05 control versus lubi and lubi versus meth

To examine time-dependent, voltage-activated hClC-2 Cl^−^ currents, a stable cell line overexpressing hClC-2 was made as described in the methods. ClC-2 is a time-dependent, voltage-activated Cl^−^ channel exhibiting inward rectification [8, 18–21] and is inhibited by CdCl_2_ [18–21]. As shown in Fig. 3a, Cl^−^ currents in HEK293EBNA cells stably expressing hClC-2 were time dependent and voltage activated, and 300 μM CdCl_2_ reduced these currents to −27.6 ± 5.7 (3) pA/pF at −140 mV, 200 ms, not significantly different than Cl^−^ currents in mock-transfected HEK293EBNA cells (see Fig. 4b). This concentration of CdCl_2_ was similar to concentrations used by others [18–21] for maximum inhibition (100–500 μM). These hClC-2 Cl^−^ currents exhibited an inwardly rectifying I–V curve, and the I–V curve became virtually linear with CdCl_2_. Although ClC-2 is described as specifically inhibited by CdCl_2_, it may also exert toxic, non-specific effects as suggested by others [8]. These control hClC-2 Cl^−^ currents were about fourfold higher than control currents measured in hClC-2-expressing HEK293 cells (Fig. 2).

### Effect of Lubiprostone on Cl^−^ Currents in hClC-2-transfected HEK293EBNA Cells

As the previous published experiments with lubiprostone [4] were carried out with hClC-2 in HEK293 cells where hClC-2 expression is low, before examining methadone and morphine effects on lubiprostone-activated hClC-2 Cl^−^ currents when expressed in HEK293EBNA cells, the effect of lubiprostone at selected concentrations was first examined, and the EC_50_ was calculated. The cells were washed with three changes of medium over about 3–4 min, in between different concentrations of lubiprostone as described previously [4]. These washes were sufficient to completely wash out the lubiprostone and return the Cl^−^ current to control levels [4]. The results are shown in Fig. 3b, plotted as current at 200 ms and −140 mV for direct comparison with previously published experiments [4]. Lubiprostone-activated hClC-2 Cl^−^ currents when expressed in HEK293EBNA cells in a concentration-dependent manner. The data were fit with a modified Michaelis–Menton equation, and the EC_50_ was 28.2 ± 2.2 nM (4), not significantly different from the EC_50_ measured for hClC-2 in HEK293 cells [4]. Also shown in the inset is the Hill plot of the data. The Hill coefficient was 0.91 ± 0.02 (4), *R* = 0.96 ± 0.09 (8), and *P* < 0.0005. These data are not significantly different from those previously reported [4]. Control- and lubiprostone-stimulated hClC-2 Cl^−^ currents in HEK293EBNA cells were about −100 and −450 pA/pF, respectively, approximately fourfold higher than control and lubiprostone-stimulated hClC-2 Cl^−^ currents expressed in HEK293 cells of about −25 and −125 pA/pF, respectively.

### Effect of Methadone on Control and Lubiprostone-stimulated Cl^−^ Currents in hClC-2- and Mock-transfected HEK293EBNA Cells

Figure 4a shows typical time-dependent, voltage-activated Cl^−^ currents (control) in hClC-2-transfected HEK293EBNA cells followed by methadone resulting in inhibition. I/V curves with methadone are also shown. Without methadone, the I/V curve was inwardly rectified, and with methadone, it was significantly inhibited (*P* < 0.01) but still slightly rectified. As shown in Fig. 4b, 20 nM lubiprostone increased hClC-2 Cl^−^ currents, and they remained time dependent and voltage activated. Subsequent addition of methadone decreased the lubiprostone-stimulated hClC-2 Cl^−^ currents. In contrast, Cl^−^ currents measured in mock-transfected HEK293EBNA cells were very low (−40.3 ± 7.6 (3) pA/pF), significantly different (*P* < 0.02) from those measured in hClC-2-transfected HEK293EBNA cells, and 20 nM lubiprostone followed by 1 μM methadone had no effect. The corresponding I/V curves for hClC-2-transfected and mock-transfected HEK293EBNA cells are also shown. Both control (without lubiprostone)- and lubiprostone-stimulated hClC-2 Cl^−^ currents in hClC-2-transfected HEK293EBNA cells showed inward rectification, while mock-transfected cells had linear I/V curves, exhibiting very small currents. Methadone caused significant decreases (*P* < 0.05) in the hClC-2 Cl^−^ current resulting in 46.2 % inhibition at −140 mV.

### Effects of Selected Concentrations of Methadone and Morphine on Cl^−^ Currents in hClC-2-Transfected HEK293EBNA Cells without (Control) and with 100 nM Lubiprostone

**Fig. 5:**
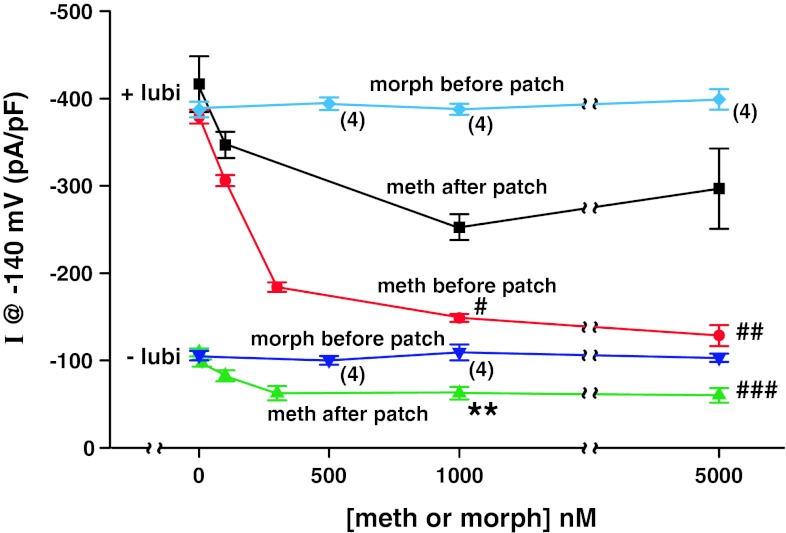
Effects of selected concentrations of methadone and morphine on Cl^−^ currents in hClC-2-expressing HEK293EBNA cells without lubiprostone (−lubi) and with lubiprostone (+lubi). hClC-2 Cl^−^ current recordings were made at selected concentrations of methadone and morphine in the absence (−lubi) or the presence (+lubi) of 100 nM lubiprostone. *I* at −140 mV and 200 ms is *plotted* as mean ± SEM (*n* = 3, except where indicated as *n* = 4). Experiments were carried out by adding compounds either before or after the cells were patched. #*P* < 0.025 and ##*P* < 0.05, both versus meth after patch. **P < 0.025 and ###*P* < 0.01 both meth after patch versus morph before patch. Concentrations of methadone resulting in half-maximal inhibition were 100 nM for control currents and 230 nM for +lubi currents meth added after patch, and 200 nM for +lubi currents meth added before patch

HEK293EBNA cells stably expressing hClC-2 without lubiprostone (control), and with 100 nM lubiprostone were treated with selected concentrations of either morphine sulfate or methadone hydrochloride added either before or after patching to measure Cl^−^ currents (Fig. 5). Lubiprostone significantly increased the Cl^−^ current, and morphine up to 5 μM had no effect on hClC-2 Cl^−^ currents without (−lubi) or with (+lubi) lubiprostone. Methadone inhibited control hClC-2 Cl^−^ currents by 45 % added after patching (shown in Fig. 5) and by 44.8 % before patching. Cl^−^ currents at −140 mV and 200 ms after and before patching were −109.2 ± 4.5 (3) and −106.6 ± 6.1 (3) pA/pF, respectively, without methadone; and −60.1 ± 8.3 (3) and −58.9 ± 2.7 (3) pA/pF, respectively, with 5 μM methadone. In contrast, methadone inhibited lubiprostone-stimulated hClC-2 Cl^−^ currents by 28.7 % when added after patching and by 66.1 % when added before patching. The methadone concentration resulting in half-maximal inhibition was 100 nM for control and 200 and 230 nM for lubiprostone-stimulated hClC-2 currents added before or after patching, respectively.

### Effect of Forskolin/IBMX, Followed by Methadone and then CdCl_2_ on Cl^−^ Currents in hClC-2-transfected HEK293EBNA Cells (A) & (B); and the Effect of the Specific PKA Inhibitor, mPKI on Forskolin/IBMX- and Lubiprostone-stimulated Cl^−^ Currents in hClC-2-Expressing HEK293EBNA Cells (C)

**Fig. 6:**
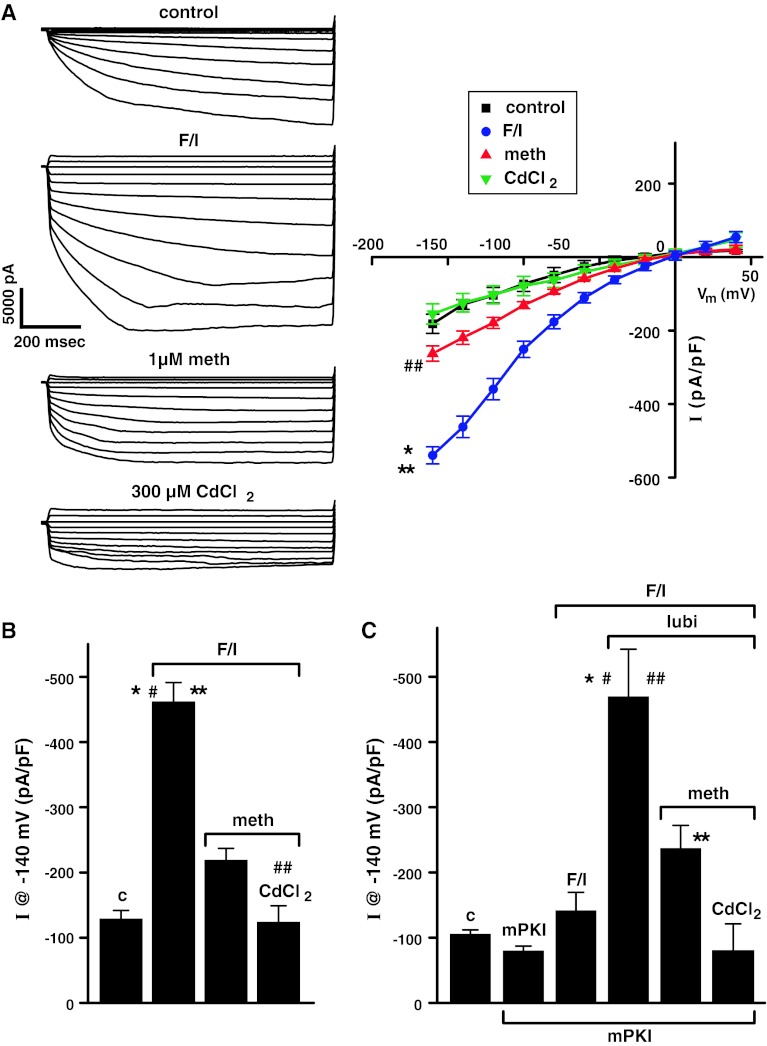
Effect of forskolin/IBMX, followed by methadone and then CdCl_2_ on Cl^−^ currents in hClC-2-transfected HEK293EBNA cells (**a**), (**b**); and (**c**) the effect of the specific PKA inhibitor, myristoylated PKI, on forskolin/IBMX- and lubiprostone-stimulated Cl^−^ currents in hClC-2-expressing HEK293EBNA cells. **a** Typical hClC-2 currents are shown before (control) and after addition of 5 μM forskolin/20 μM IBMX, followed by 1 μM methadone and then 300 μM CdCl_2_ (cell capacitance = 32.6 pF). Corresponding I/V *curves* are also shown expressed as *I* at 200 ms. Data are *plotted* as mean ± SEM, *n* = 3. *P < 0.001 versus meth, **P < 0.0005 versus control and CdCl_2_, ##*P* < 0.025 versus CdCl_2_, F/I versus meth −140 to −60 mV *P* < 0.005–0.05; F/I versus CdCl_2_ −140 to −60 mV *P* < 0.0005–0.025. **b** Cl^−^ currents (at 200 ms and −140 mV) in hClC-2-expressing HEK293EBNA cells before (control, c) and after 5 μM forskolin/20 μM IBMX (F/I) addition, followed by 1 μM methadone (meth), and followed by CdCl_2_, are *plotted* as mean ± SEM, *n* = 3. *P < 0.001 versus control, #*P* < 0.005 versus meth, **P < 0.0005 versus CdCl_2_, ##*P* < 0.02 versus meth. **c** Cl^−^ currents (at 200 ms and −140 mV) in hClC-2-expressing HEK293EBNA cells before (control, c) and after 0.4 μM mPKI addition, followed by sequentially adding 5 μM forskolin/20 μM IBMX, 100 nM lubiprostone, 1 μM methadone, and finally 300 μM CdCl_2_ are *plotted* as mean ± SEM, *n* = 3 *P < 0.01 versus F/I, mPKI and c, #*P* < 0.05 versus meth, ##*P* < 0.005, and **P < 0.025 both versus CdCl_2_

The effect of 5 μM forskolin/20 μM IBMX, followed by 1 μM methadone and then 300 μM CdCl_2_ on Cl^−^ currents in hClC-2-transfected HEK293EBNA cells was investigated, and the results are shown in Fig. 6. Figure 6a shows typical time-dependent, voltage-activated hClC-2 Cl^−^ currents stimulated by forskolin/IBMX, inhibited by 1 μM methadone and further inhibited by 300 μM CdCl_2_. The corresponding I/V curves are also shown, and they were all inwardly rectified, even after inhibition. The effect of the specific PKA inhibitor, mPKI, was then investigated. Figure 6b, c show the effects of forskolin/IBMX, methadone, and CdCl_2_ on hClC-2 Cl^−^ currents in the absence and the presence of 0.4 μM mPKI, respectively. As shown in Fig. 6b, forskolin/IBMX (5/20 μM) significantly stimulated hClC-2 Cl^−^ currents (*P* < 0.001), 1 μM methadone significantly inhibited this response (*P* < 0.005), and CdCl_2_ (300 μM) further inhibited the Cl^−^ currents (*P* < 0.02). In contrast, as shown in Fig. 6b, forskolin/IBMX had no effect on hClC-2 Cl^−^ currents in the presence of 0.4 μM mPKI. However, 100 nM lubiprostone stimulated hClC-2 Cl^−^ currents significantly (*P* < 0.01) even in the presence of mPKI. This mPKI-insensitive, lubiprostone-stimulated Cl^−^ current was inhibited by 1 μM methadone (*P* < 0.05) and further inhibited by 300 μM CdCl_2_. (*P* < 0.025).

## Discussion

Lubiprostone is very effective in treating opioid-induced constipation induced by morphine and congeners [1–3]. The inhibitory effect of methadone on lubiprostone-mediated relief from constipation [3] may arise from methadone inhibition of ClC-2 Cl^−^ currents. The present study was designed to test the hypothesis whether methadone, but not morphine, might inhibit Cl^−^ transport by epithelial cells. Lubiprostone-stimulated Cl^−^ currents measured by Isc in T84 cells and control and lubiprostone-stimulated hClC-2 Cl^−^ currents were inhibited by methadone, but not by morphine. The half-maximal concentration for methadone inhibition of Cl^−^ currents measured by Isc was 100 nM, approximately 18 times the affinity of methadone for mu receptors [34]. Naloxone alone, or with methadone, had no effect. Morphine, even at 5 μM (2,500 times higher concentration than its affinity for mu receptors) [34], had no effect. Therefore, inhibition by methadone of T84 cell lubiprostone-stimulated Isc appears to be consistent with the reduced effect of lubiprostone on methadone-induced constipation [3].

Methadone and morphine both bind to mu receptors on target cells [34, 35], although they belong to two different classes of opioids distinct in chemical structure (diphenylheptanes vs. phenanthrenes) and metabolic pathways. In the intestine, mu receptors have been localized largely to nerve terminals and synaptic elements in all intestinal layers [11, 12]. Although human colonocytes have been suggested to have mu receptors [33], no evidence for mu receptors on intestinal epithelial cells was found by others [11, 12, 31, 32]. Lubiprostone stimulates Cl^−^ transport across epithelial cells [4–6]. This raised questions regarding the mechanism whereby the beneficial lubiprostone effects could be affected by methadone, but not morphine in the clinical situation. The reported finding of methadone, but not morphine attenuating/preventing the beneficial effects of lubiprostone in the clinical trial [3] was difficult to relate to mu receptors, as both agents bind mu receptors with similar affinity (EC_50_): 5.6 nM for methadone and 2.0 nM for morphine, tested with the cloned human mu receptor [34]. Thus, methadone might interfere with lubiprostone action by a mechanism independent of mu receptor binding.

The finding of methadone inhibition of T84 cell lubiprostone-stimulated Cl^−^ currents measured by Isc was unexpected, as there are no reports of effects of methadone on Cl^−^ currents in the literature. If mu receptors were not involved in the process of inhibition by methadone, as suggested by the lack of naloxone effect, then some other target required for Cl^−^ current activation might be affected. Direct action on ion channels or a process required for activation of ion channel function might be responsible for methadone inhibition. Methadone, but not morphine, inhibits l-type calcium channels [13], various potassium channels [14, 15], and hERG [16, 17]. Direct inhibition of hERG might underlie cardiotoxicity seen with methadone treatment. In the latter case, methadone binds largely to the inactivated/open channel, and the inhibitory effect occurs within 10 ms. Methadone has been suggested to act through binding to the voltage sensor of hERG [16, 17]. These findings provided a basis for examination of whether methadone affected the function of either recombinant hClC-2 or hCFTR.

Both hClC-2 and hCFTR ion channels have been suggested to be involved in lubiprostone stimulation of T84 cell Isc [4–6], but have not been directly identified. A similar concentration dependence for lubiprostone stimulation of recombinant hClC-2 and T84 cell Isc was reported, while no stimulation of hCFTR was observed at concentrations as high as 1 μM lubiprostone [4]. In single-channel studies, A6 cell ClC-2 as well as hClC-2 expressed in HEK293 cells were activated by lubiprostone at low concentrations (<100 nM), while CFTR was also activated by lubiprostone, but at concentrations 50 times higher than the concentration necessary to activate ClC-2 [8]. Knockdown of T84 cell ClC-2 ablated lubiprostone stimulation of T84 cell Cl^−^ currents [7]. Based on T84 cell Isc studies [5, 6] and mouse intestine Isc studies [5] in the presence and absence of CFTRinh172, ClC-2 has been suggested to not be involved in lubiprostone stimulation, but rather CFTR. In those studies, lubiprostone stimulation of Isc was inhibited by CFTRinh172, but it is unknown whether CFTRinh172 also inhibits ClC-2. CFTRinh172 did not inhibit Ca^2+^-activated Cl^−^ currents in human airway cells or volume-activated Cl^−^ currents in Fischer rat thyroid cells [36] and these were the only Cl^−^ channels tested. Until CFTRinh172 has been shown to not inhibit ClC-2, it is difficult to evaluate whether ClC-2 or CFTR was being activated by lubiprostone in those studies.

Studies of effects of methadone on hClC-2 and hCFTR were undertaken. Previously established HEK293 cell lines stably transfected with hClC-2 or hCFTR [4], and a newly developed cell line, HEK293EBNA transfected with hClC-2 were used for studies of lubiprostone stimulation of hClC-2 and the effects of opioids. The newly developed cell line overexpressed hClC-2, leading to higher control Cl^−^ currents (approximately −100 pA/pF compared to approximately −25 pA/pF), allowing studies of the effects of lubiprostone and opioids on the time-dependent, voltage-activated hClC-2 Cl^−^ currents, infrequently exhibited by our previously used hClC-2 in HEK293 cell line [4, 9, 10]. The reason(s) for this infrequent time dependence and voltage activation is(are) not known. However, hClC-2 Cl^−^ currents in parental human cystic fibrosis airway, IB3-1, cells were shown to lack time dependence and voltage activation, but subsequent overexpression of hClC-2 in IB3-1 cells resulted in time-dependent, voltage-activated Cl^−^ currents [19].

Lubiprostone-stimulated hClC-2 Cl^−^ currents were inhibited by methadone, but not morphine. Addition of methadone before or after patching altered the extent, but not the concentration of methadone giving half-maximal inhibition of lubiprostone-stimulated hClC-2 Cl^−^ currents. Methadone inhibition was greater when added before patching, suggesting that methadone does not bind to activated or open hClC-2 Cl^−^ channels. Determination of the basis for this effect is beyond the scope of the present study, and this effect probably has no therapeutic implications as methadone is maintained throughout treatment. Methadone inhibition also occurred in the presence of morphine. Methadone was found to be effective in inhibiting lubiprostone and forskolin/IBMX-stimulated hClC-2 Cl^−^ currents whether morphine was also present or not. The lack of effect of methadone on Cl^−^ currents in hCFTR-transfected HEK293 cells suggested inhibitory methadone effects were related to hClC-2 or the processes involved in the activation of lubiprostone-stimulated hClC-2 Cl^−^ currents.

Mu receptors do not appear responsible for methadone inhibition of recombinant hClC-2 or T84 Cl^−^ currents based on the high concentrations of methadone required for inhibition compared with its affinity for mu receptors, and the lack of effect of morphine. HEK293 cells appear unlikely to have mu receptors as judged by lack of specific mu receptor binding and lack of mu receptor protein detected by immunoblot [37] and have been widely used to study recombinant mu opioid receptors [38, 39]. HEK293 cells expressing recombinant ion channels are therefore suitable for studies of effects of methadone and morphine. Intestinal epithelia have been variously reported to lack opiate receptors [11, 12, 31, 32], or have mu receptors on human colonocytes [33]. There are no reports on whether T84 cells themselves have mu receptors. However, there was no effect of naloxone [13] on methadone inhibition of lubiprostone-stimulated Cl^−^ currents in T84 cells.

Using a newly developed cell line (HEK293EBNA transfected with hClC-2) exhibiting time-dependent, voltage-activated Cl^−^ currents, methadone, but not morphine, inhibited these hClC-2 Cl^−^ currents. Thus, methadone does not act by competition with lubiprostone, suggesting possible interaction between methadone and hClC-2 (or a closely related process required for activation of hClC-2 dependent Cl^−^ currents). Further studies will be required to determine if hClC-2 interacts directly with methadone.

Inhibitory effects of methadone on forskolin/IBMX activated Cl^−^ currents were also observed in hClC-2-expressing HEK293EBNA cells. This effect was prevented by the PKA-specific inhibitor, mPKI. Thus, forskolin/IBMX stimulation appeared to be through PKA activation of hClC-2, as previously demonstrated in functional and site-directed mutagenesis studies [4, 10]. Lubiprostone-stimulated hClC-2 Cl^−^ currents were unaffected by mPKI. Thus, inhibition of hClC-2 by methadone does not appear to be through competition with lubiprostone, consistent with the possibility of direct binding of methadone to hClC-2. Alternatively, methadone might interfere with a process blocking Cl^−^ currents in general (without directly affecting the ClC-2 channel protein). However, methadone was without effect on hCFTR Cl^−^ currents. Therefore, methadone is apparently not affecting a process important to Cl^−^ transport, per se, but might interact with either ClC-2 or a process required for transport of Cl^−^ by ClC-2.

Single-channel studies of hClC-2 have been published using the same HEK293 cells transfected with hClC-2 as used in Fig. 2 [8]. In Fig. 17 of this published article, the authors state referring to HEK293 cells expressing hClC-2: “the anion channel activated by lubiprostone had channel kinetics and a current–voltage relationship that were essentially indistinguishable from the channels in A6 cells we had identified as ClC-2 channels.” It is not clear whether single-channel studies of methadone effects on hClC-2 would lead to further understanding of the mechanism.
